# Anti-Inflammatory Effect of Cherry Extract Loaded in Polymeric Nanoparticles: Relevance of Particle Internalization in Endothelial Cells

**DOI:** 10.3390/pharmaceutics11100500

**Published:** 2019-09-29

**Authors:** Denise Beconcini, Francesca Felice, Ylenia Zambito, Angela Fabiano, Anna Maria Piras, Maria Helena Macedo, Bruno Sarmento, Rossella Di Stefano

**Affiliations:** 1 Department of Life Sciences, University of Siena, via P.A. Mattioli 4, 53100 Siena, Italy; denisebeconcini@gmail.com; 2 Cardiovascular Research Laboratory, Department of Surgery, Medical, Molecular, and Critical Area Pathology, University of Pisa, via Paradisa 2, 56100 Pisa, Italy; rossella.distefano@unipi.it (R.D.S.); francesca.felice77@hotmail.it (F.F.); 3 Department of Pharmacy, University of Pisa, via Bonanno 33, 56100 Pisa, Italy; angela.fabiano@unipi.it (A.F.); anna.piras@unipi.it (A.M.P.); 4 Interdepartmental Research Center Nutraceuticals and Food for Health, University of Pisa, via Borghetto 80, 56100 Pisa, Italy; 5 i3S-Instituto de Investigação e Inovação em Saúde, University of Porto, Rua Alfredo Allen 208, 4200-153 Porto, Portugal; helena.macedo@i3s.up.pt; 6 INEB—Instituto de Engenharia Biomédica, Universidade do Porto, Rua Alfredo Allen, 208, 4200-135 Porto, Portugal; 7 ICBAS—Instituto de Ciências Biomédicas Abel Salazar, University of Porto, Rua de Jorge Viterbo Ferreira 228, 4050-313 Porto, Portugal; 8 CESPU, Instituto de Investigação e Formação Avançada em Ciências e Tecnologias da Saúde, Rua Central de Gandra, 1317, 4585-116 Gandra, Portugal

**Keywords:** inflammation, sweet cherry (*Prunus avium* L.), polyphenols, nanoparticles, HUVEC, NLRP3 inflammasome

## Abstract

This study aimed at evaluating the anti-inflammatory effect of natural cherry extract (CE), either free or encapsulated in nanoparticles (NPs) based on chitosan derivatives (Ch-der) or poly(lactic-*co*-glycolic acid) (PLGA), on human umbilical vein endothelial cells (HUVEC). CE from *Prunus avium* L. was characterized for total polyphenols, flavonoids, and anthocyanins content. CE and CE-loaded NP cytotoxicity and protective effect on lipopolysaccharide (LPS)-stressed HUVEC were tested by water-soluble tetrazolium salt (WST-1) assay. Pro- and anti-inflammatory cytokines (TNF-α, IL-6, IL-10, and PGE2) released by HUVEC were quantified by enzyme-linked immunosorbent assay (ELISA). All NP types were internalized into HUVEC after 2 h incubation and promoted the anti-inflammatory effect of free CE at the concentration of 2 µg gallic acid equivalents (GAE)/mL. CE-loaded Ch-der NPs showed the highest in vitro uptake and anti-inflammatory activity, blunting the secretion of IL-6, TNF-α, and PGE2 cytokines. Moreover, all NPs reduced the production of nitric oxide and NLRP3 inflammasome, and had a stronger anti-inflammatory effect than the major corticosteroid dexamethasone. In particular, the results demonstrate that natural CE protects endothelial cells from inflammatory stress when encapsulated in NPs based on quaternary ammonium chitosan. The CE beneficial effects were directly related with in vitro internalization of CE-loaded NPs.

## 1. Introduction

Cardiovascular risk factors, such as smoking, arterial hypertension, dyslipidaemia, diabetes mellitus, and obesity, are the main causes of endothelial dysfunction and consequent inflammatory response that may promote the development of atherosclerosis. Recently, the activation of the NLRP3 (nucleotide-binding domain and leucine-rich repeat receptor containing a pyrin domain 3) inflammasome and the oxidative stress caused by dysfunctional mitochondria, immune cell dysregulation, and chronic infections have shown a pivotal role in atherosclerosis and inflammaging [1,2]. This condition is characterized by high levels of blood inflammatory markers, which leads to high susceptibility to chronic morbidity, disability, frailty, and premature death [1,2]. Calder et al. [3] demonstrated the key role of nutrition in inflammaging and the importance of a healthy Mediterranean diet, based on fruit and vegetables, as a possible alternative strategy for reducing inflammation and related inflammatory factors.

Several clinical studies have demonstrated that cherry fruit or cherry juice consumption can prevent or reduce inflammation related to muscle damage caused by intensive strength exercise [4,5,6,7], and also accelerate the recovery following strenuous physical activity [8]. Moreover, it was shown that tart cherry concentrate attenuates oxidative stress and inflammation in an exercise task that induces stress almost exclusively from metabolic pathways [9]. All of these advantages are attributed to the vascular smooth muscle cells uptake of anti-inflammatory and antioxidant phytochemicals contained within the juice [10], and to the positive effects of polyphenols-rich cherries on vascular function [11]. Other studies have clearly proved that flavonoids and anthocyanins, such as cyanidin-3-o-glucoside and quercetin, in autochthonous cherry cultivars are responsible for inhibiting lipopolysaccharide (LPS)-induced inflammation and favoring the release of endothelial-derived vasoactive factors after vascular endothelial damage [12,13,14,15,16]. A recent study has confirmed the importance of cherry fruit in atherosclerosis risk factors prevention, and the main role of polyphenols in inflammation reduction and endothelial dysfunction improvement [17].

However, most of the works cited above tested commercial or supplemented cherry juice, whereas only a few studies about cherry anti-inflammatory in vitro activity have been reported. In fact, none deal with cherry extract effect on endothelial cells, despite the direct involvement of these cells in endothelial dysfunction. As it is known, however, the antioxidant and anti-inflammatory molecules contained in fruit once ingested are readily degraded in the gastrointestinal tract; therefore, their oral bioavailability is always very low.

On the basis of our previous studies [18,19,20,21], the encapsulation of cherry extract in nanoparticles (NPs) may enhance the polyphenols’ oral bioavailability and improve their health effects. For these reasons, encapsulation of autochthonous natural sweet cherry extract (CE) from *Prunus avium* L. in NPs was chosen to study the beneficial effects of CE polyphenols against oxidative stress and inflammation, as well as NLRP3 inflammasome activation. Human umbilical vein endothelial cells (HUVEC) were used as the substrate. In particular, the anti-inflammatory effect on LPS-stressed HUVEC, and the production of pro- and anti-inflammatory factors and NLRP3, were evaluated. To understand the relevance of CE-loaded NP characteristics to their anti-inflammatory activity and their cellular uptake, the studies were carried out with CE polyphenols either free or encapsulated in NPs based on different polymer types, i.e., two different quaternary ammonium chitosan derivative (Ch-der) NPs or poly(lactic-*co*-glycolic acid) (PLGA) NPs [20].

## 2. Materials and Methods

HUVEC derived from the endothelium of veins of healthy human donor umbilical cords [22] were used for in vitro studies. They were cultured in gelatin pre-coated flasks in complete EGM-2 Endothelial Medium BulletKit (EuroClone S.p.A., MI, Italy).

For NP preparation, PLGA 5004 A (50:50, molecular weight (MW) ≈ 44 kDa), offered by Corbion-Purac Biomaterials (Gorinchem, AC, Netherlands), ethyl acetate (EA), and Kolliphor^®^ P 407 (Sigma-Aldrich, St. Louis, MO, USA) were used for PLGA NP preparation. Ch-der NPs were prepared from the following polymers: Quaternary ammonium chitosan (QA-Ch) conjugate synthesized at 60 °C from low-molecular-weight Ch (Sigma, Darmstadt, Germany) [23] (NP1); thiolated derivative of QA-Ch [24] with protected thiols, coded QA-Ch-S-pro [25] (NP2); reduced-MW hyaluronic acid rHA, viscosimetric MW 470 kDa, from HA MW 950 kDa (Contipro, Dolní Dobrouč, Czech Republic) [26]. Fluorescein isothiocyanate (FITC) and dimethyl sulfoxide, purchased from Sigma-Aldrich (St. Louis, MO, USA), were used for the FITC-labeling of PLGA, QA-Ch, and QA-Ch-S-pro polymers.

Hank’s balanced salt solution (HBSS 1× without phenol red) (Gibco Waltham, MA, USA), CellMask™ Orange Plasma Membrane Stain (Life technologies, Carlsbad, CA, USA), 4,6-Diamidino-2-phenylindole (DAPI), and paraformaldehyde (PFA) (Electron Microscopy Sciences, Hatfield, UK) were used in in vitro uptake studies.

4-[3-(4-iodophenyl)-2-(4nitrophenyl)-2*H*-5-tetrazolium]-1,3-benzene disulfonate (WST-1), provided by Roche Applied Science (Mannheim, Germany), and LPS and dexamethasone (DEXA) from Sigma-Aldrich (St. Louis, MO, USA), were used in cell viability studies.

Milli-Q^®^ water, phosphate buffer pH 7.4 (PB, 0.065 M), and phosphate-buffered saline (PBS) for cell culture were homemade.

### 2.1. Fruit Material and NP Preparation

Crognola Capannile cherry fruits (*Prunus Avium* L.) were harvested at their highest degree of maturation (1 June–25 July) from plants present in the Tuscan germplasm collection of the Santa Paolina experimental farm, which is part of the National Research Council of Italy Trees and Timber Institute (CNR IVALSA) located in Follonica (Italy). The cherry extracts were characterized for polyphenols, flavonoids, and anthocyanins total content, as well as antioxidant potential, as previously described [21,27]. Briefly, the total polyphenol content (TPC) of CE samples, before and after freeze-drying, was determined by the spectrophotometric method of Folin–Ciocalteau [28]; flavonoids and anthocyanins total content were quantified with the aluminum chloride method [29] and the pH differential spectroscopic method [30], respectively; the antioxidant potential of CE freeze-dried samples was determined using the ferric-reducing antioxidant power (FRAP) assay reported by Benzie and Strain [31]. The presence of specific secondary metabolites in Crognola, such as chlorogenic acid, *p*-coumaric acid, (+)-catechin, rutin, and cyanidin-3-glucoside (CY3G), was also quantified by the HPLC method [27].

Both empty and CE-loaded Ch-der or PLGA NP preparation and characterization have already been reported in our previous works [20,21]. Briefly, to prepare CE-loaded NPs based on QA-Ch and QA-Ch-S-pro, a 650-µL volume of PB, containing 0.05 mg/mL rHA and 4.5 mg/mL CE, was added portion-wise (50 µL) to 5 mL of 0.5 mg/mL Ch derivative solution in PB under stirring [21]. PLGA NPs were prepared through a w/o/w double emulsion technique [20]: 100 mg PLGA 50:50 was dissolved in 2 mL of EA overnight; then, 100 µL of CE (30 mg/mL, 840 µg/mL gallic acid equivalents (GAE) solution in water) was added, and the first emulsion was obtained after vortexing. To stabilize this emulsion, 4 mL of the surfactant solution, 0.5% Kolliphor^®^ P 407 in water, was added, then the emulsion was homogenized for 1 min with a Vibra-Cell™ ultrasonic processor (amplitude, 70%). Finally, the second emulsion (w/o/w) was added into 8 mL of the same surfactant solution. This emulsion was left for 3 h in a fume hood under magnetic stirring at 300 rpm for EA evaporation. All of the NPs were characterized for size, polydispersity index, and zeta potential (ζ) using a Malvern Zetasizer Nano ZS instrument (Malvern Instruments Ltd., Worcestershire, UK).

The FITC-labeling of PLGA, QA-Ch, and QA-Ch-S-pro polymers was previously described [20,27]. Empty FITC-labeled NPs were produced in the same way as non-FITC-labeled NPs. After preparation, FITC-labeled NPs were characterized for size, polydispersity index, and zeta potential using a Malvern Zetasizer Nano ZS instrument (Malvern Instruments Ltd., Worcestershire, UK).

### 2.2. Experimental Protocol

HUVEC were cultured in EGM-2 complete medium for one week and used for experiments (passages 5 to 7). Cells (1.5–2 × 10^4^ per well) were seeded into 96-well plates (Becton Dickinson, San Jose, CA, USA) and incubated for 24 h. Then, cells were incubated for 2 h with free CE diluted to the polyphenols concentrations of 2, 5, or 10 μg GAE/mL culture medium (M199 5% FBS). Also, HUVEC were incubated for 2 h with different types of empty or CE (2 μg/mL GAE)-loaded NPs based on Ch-der (QA-Ch or QA-Ch-S-pro) or PLGA NPs. The concentration of 2 μg/mL GAE was the maximum non-toxic load previously tested [20,21]. The NPs were freshly prepared and diluted in culture medium to the desired concentrations. To study the anti-inflammatory effect of loaded NPs, DEXA (5 μg/mL culture medium) was used as positive control. After a 2-h incubation, cells were washed twice with PBS and treated with LPS (10 μg/mL) for 24 h to induce inflammatory stress. At the end of the treatments, the supernatants were collected and stored at −80° C for further analysis. Cell viability, before and after LPS treatment, was measured using the WST-1 assay, and the values obtained were compared with 100% of control (untreated cells). All treatments were done in triplicate and all experimental data resulted from at least two triplicate runs.

### 2.3. Measurement of Inflammatory Cytokines Production

After cell treatments, anti-inflammatory activity was evaluated. Briefly, the cytokines (IL-6, IL-10, PGE2, TNF-α) released from cells in the supernatants after CE, free NP, or CE-loaded NP pre-treatments and LPS inflammatory stress induction were measured. IL-10 and IL-6 were quantified by enzyme-linked immunosorbent assay (ELISA) (Invitrogen™ Human IL-10 ELISA Kit and Invitrogen™ Human IL-6 ELISA Kit, Vienna, Austria). The amount of PGE2 was quantified by competitive immunoassay (Invitrogen™ Human Prostaglandin E2, Camarillo, CA, USA). Tumor necrosis factor alpha (TNF-α) production was determined by a solid phase enzyme amplified sensitivity immunoassay (BIOSOURCE TNF-α EASIA kit, Nivelles, Belgium).

### 2.4. NLRP3 Inflammasome Production

A sandwich enzyme immunoassay (Enzyme-linked Immunosorbent Assay Kit For NLR Family, Pyrin Domain Containing Protein 3 (NLRP3), (Cloud-Clone, Katy, TX, USA) was used for the quantitative measurement of NLRP3 inflammasome in HUVEC supernatants.

### 2.5. Nitric Oxide Assay

Both nitrite and nitrate production after inflammatory stress was assayed by a Nitric Oxid Assay Kit (Invitrogen™, Frederick, Maryland, MD, USA). The concentrations of total nitrite [(NO_2_)^−^]_tot_ derived from nitrate [(NO_3_)^−^] reduction. The nitrate concentration was calculated by subtracting the nitrite concentration [(NO_2_)^−^] from the total nitrite concentration, obtained by Nitrite Assay, as follows:

[(NO_3_)^−^] = [(NO_2_)^−^]_tot_ − [(NO_2_)^−^] (1)

Since the endogenous nitrite concentration was almost zero, the nitrite concentration in supernatant was strictly dependent on nitrate reduction. Both nitrate and total nitrite concentrations were expressed as µmol/L.

### 2.6. In Vitro Uptake Studies

To study Ch-der and PLGA NPs in vitro uptake, both HUVEC and C2BBe1 (see Figure S1) were seeded in 24-well plates (2 × 10^5^ cells/well) on glass coverslips with complete culture medium and incubated for 24 h. Afterwards, cells were washed with PBS and treated for 1 h or 2 h with freshly prepared FITC-labeled Ch-der NPs or PLGA NPs diluted in HBSS to the polymer concentrations of 0.05 mg/mL or 2.4 mg/mL, respectively. Those were the maximum non-cytotoxic concentrations previously tested [20]. Cells with HBSS only were used as control. After treatment, the supernatant was discarded and cells were washed twice with PBS. A previous protocol [32] was adapted for the subsequent steps.

### 2.7. Confocal Laser Scanning Microscopy (CLSM)

After treatment and washing, carried out as described in Section 2.6., cells were stained with CellMask™ Orange Plasma Membrane Stain (dilution 1:20 in PBS) for 5 min at 37 °C, fixed with PFA 2% for 15 min, and then stained with DAPI (100 ng/mL) 15 min at room temperature in the dark. A double washing with PBS followed each passage. At the end, coverslips were mounted onto clean slides and maintained at 4 °C in the dark. The analysis was made by Spectral Confocal Microscope Leica TCS-SP5 AOBS, Leica Microsystems (Wetzlar, Germany) using a 63× HCX PL APO CS oil objective and performing Z stacks (zoom of 1.0). Images were acquired by setting the pinhole at 95.5 μm, the image size of 512 × 512 pixels, 8 bits of resolution and a bidirectional scanning speed at 400 Hz. DAPI, FITC-labeled NPs, and the CellMask™ Orange Plasma Membrane Stain were excited by a 405 Diode UV, an argon, and a DPSS 561 laser, respectively. Untreated cells were used as a control to adjust the gain of each detector and remove the green autofluorescence of the cells. Using the same adjusted settings, a Z-stack of all treated samples was performed with a step size of 0.5 µm (20–30 slices per each image). Images were treated through ImageJ 1.48 V Software.

### 2.8. ImageStream^®^X Imaging Flow Cytometer

HUVEC and C2BBe1 (see Figure S2) were seeded in 6-well plates (3 × 10^5^ cells/well) and treated as described in Section 2.6. Then, cells were detached from the well through the use of trypsine and each collected suspension was centrifuged (1100 rpm, 6 min, RT); the pellet was resuspended in 300 μL of PBS and centrifuged again to remove medium. After that, cells were resuspended in PFA 2%, following 20 min of incubation at room temperature. After a new centrifugation step to remove PFA, the supernatant was discarded again and cells were resuspended in 100 μL of fresh PBS for further analysis. After filtration through a nylon mesh, all samples were analyzed through the ImageStream^®^X imaging flow cytometer equipped with INSPIRE^®^ software (Amnis Corporation, Seattle, WA, USA). Intensity-adjusted brightfield images of the cells were collected and the fluorescent NPs were analyzed through an excitation laser at 488 nm with intensity of 10 mW. Untreated cells were also analyzed for control to evaluate the autofluorescence of cells. For each sample, 500–1000 cells were analyzed. The fluorescence intensity of each cell was analyzed through the use of the internalization wizard provided by the IDEAS^®^ v6.2 software (Amnis Corporation, Seattle, WA, USA). The internalization score was achieved after the generation of an erode mask with 4 pixels on the whole cell (brightfield channel), which allowed the elimination of fluorescence signal caused by adsorbed NPs on the cell surface, and consequently only the fluorescence intensity ascribable to internalized NPs was quantified [32,33]. Cells with an IS score higher than 1 were gated and the fluorescence intensity of each cell was acquired.

### 2.9. Flow Cytometry and Fluorescence-Activated Cell Sorting (FACS)

FACS analysis was performed according to a protocol previously described [34]. C2BBe1 (Figure S3) and HUVEC were seeded at a density of 2 × 10^5^ cells per well in 12-well plates, and allowed to attach overnight at 37 °C. The medium was then removed and 1 mL of FITC-labeled NPs dispersed in HBSS was added to each well and incubated for 2 h at 37 °C. Afterward, the NP suspension was removed and the wells were washed twice with PBS (pH 7.4) to remove non-associated NPs. Next, the cells were detached using trypsin solution, centrifuged, and resuspended in PBS (300 µL, pH 7.4) to immediately measure the cellular associations using Accuri™ C6 Flow Cytometer (BD Biosciences, San Jose, CA, USA). To detect the amount of NPs internalized into the cells, trypan blue 0.005% *v/v* was used to quench the fluorescence of NPs attached to the surfaces of the cell membranes. Briefly, cells were incubated 4 min with trypan blue, centrifuged, and resuspended again in PBS (300 µL, pH 7.4). All data were analyzed with FlowJo software (Tree Star, Inc., Ashland, OR, USA).

### 2.10. Statistical Analysis

The GraphPad Prism Software version 7.0 (GraphPad Software Inc., La Jolla, CA, USA) was used for the statistical analysis of data. All values were tested for normal distribution and equal variance. When homogeneous variances were confirmed, data were analyzed by one-way analysis of variance (ANOVA) coupled with the post-hoc Dunnett test to identify means with significant differences. Paired comparisons were done by Student’s *t*-tests. Differences were considered significant, i.e., the null hypothesis was rejected, for *p*-values lower than 0.05. All results were presented as means ± standard deviation of at least three independent experiments.

## 3. Results

### 3.1. Cherry Extract Characterization

The TPC of CE before and after freeze drying were 402.5 ± 8.4 mg GAE/100 g fresh weight (FW) and 26.7 μg/mL GAE per milligram of dry weight, respectively [21]; while the antioxidant potential was 0.229 mg of Fe^2+^/mL [21]. The total content of flavonoids and anthocyanins in Crognola was the highest in the sweet cherry varieties studied by Berni et al. [27], namely 81.6 mg quercetin equivalents (QuE) and 67.8 mg cyanidin-3-glucoside equivalents (CyE) per 100 g of FW, respectively. Among anthocyanins, CY3G, which is responsible for inhibiting lipopolysaccharide (LPS)-induced inflammation [14], resulted the most relevant component (151.2 μg/g FW). These values show that sweet cherries were one of the most abundant anthocyanins-rich red fruit [27] and that autochthonous fruit contained many functional molecules, in many cases even more than those found in commercial fruit [35]. To confirm the beneficial effects of Crognola CE, we evaluated the anti-inflammatory potential of CE anthocyanins through in vitro studies.

### 3.2. FITC-Labeled NP Characterization

The physical characteristics of empty FITC-labeled NPs used in internalization studies are listed in Table 1. While size, polydispersity index, and zeta potential (ζ) for fluorescent PLGA NPs remained about the same as non-fluorescent PLGA NPs [20], a significant reduction of all these parameters was seen in FITC-labeled Ch-der NPs, when compared with the non-FITC-labeled Ch-der NPs [21]. Since we never found any significant differences in these parameters between FITC-labeled and unlabeled NPs obtained with the same Ch-der in the past [19], this difference may be due to the CE encapsulated in NPs. In fact, particle size (NP1: 344.9 ± 17.8; NP2: 339.9 ± 68.2; PLGA NPs: 206.1 ± 1.8), polydispersity index (NP1: 0.52 ± 0.08; NP2: 0.50 ± 0.09; PLGA NPs: 0.06 ± 0.03), zeta potential (NP1: 14.8 ± 0.3; NP2: 15.8 ± 0.5; PLGA NPs: −8.36 ± 1.07), and entrapment efficiency % (NP1: 78.4 ± 4.5; NP2: 79.8 ± 0.6; PLGA NPs: 88.6 ± 6.2) of CE-loaded NPs were measured in our previous works [20,21].

### 3.3. Cell Viability

**Figure 1:**
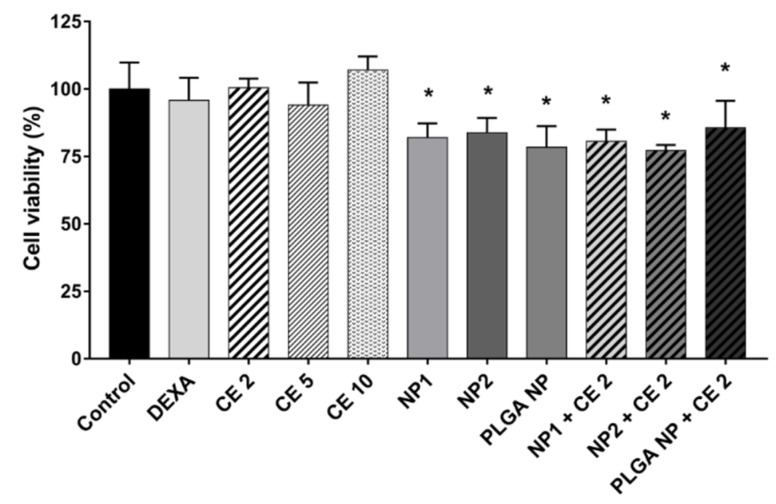
Cell viability. Human umbilical vein endothelial cells (HUVEC) were treated with different cherry extract (CE) polyphenols concentrations (2, 5, 10 µg gallic acid equivalents (GAE)/mL) for 2 h and with empty or CE-loaded NPs (NP1: QA-Ch-NP; NP2: QA-Ch-S-pro-NP; and PLGA NPs) with 2 µg GAE/mL of CE (CE 2) or dexamethasone (DEXA) (5 µg/mL) for 2 h. Data are the means ± standard deviation (SD) of three independent experiments. * *p* < 0.05 vs. Control (untreated cells).

The cell viability of various concentrations of CE (2–10 µg GAE/mL) and different nanoparticles (QA-Ch-NP: NP1; QA-Ch-S-pro-NP: NP2; and PLGA NP) free or loaded with 2 µg/mL GAE of CE, already determined by WST-1 colorimetric assay in our previous paper [20], was investigated again to verify the reproducibility of the results obtained with materials from different lots and compare them directly with those obtained in the presence of LPS, never studied before. Figure 1 shows no cytotoxic effect of DEXA or CE at different concentrations. A cell viability reduction was observed in the presence of empty or loaded NPs compared to control. However, cell viability was not less than 77% (* *p* < 0.05 vs. Control), as we had already seen in our previous work [20].

**Figure 2:**
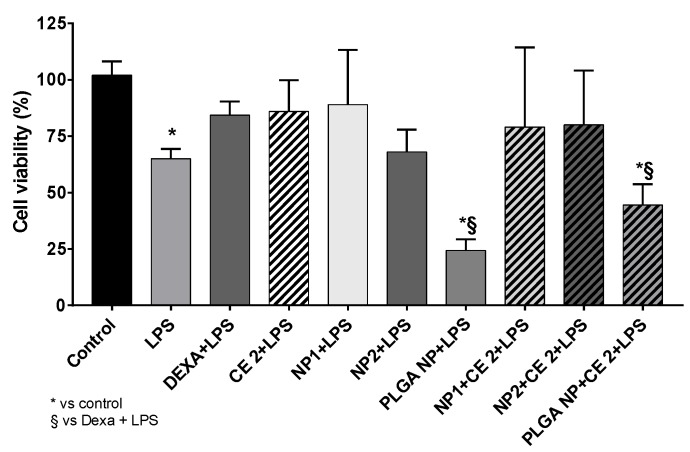
Cell viability. HUVEC were treated with empty or CE-loaded NPs (QA-Ch-NP: NP1; QA-Ch-S-pro-NP: NP2; and PLGA NPs), with 2 µg/mL GAE of CE (CE 2) or DEXA (5 µg/mL) for 2 h and with lipopolysaccharide (LPS) (10 µg/mL) for 24 h. Data are the means ± SD of three independent experiments. * *p* < 0.05 vs. Control and § *p* < 0.05 vs. DEXA + LPS.

The presence of LPS significantly reduced cell viability (Figure 2). Moreover, cells pre-treated with empty or CE-loaded PLGA NPs for 2 h and with LPS for 24 h showed a significant reduction of cell viability (* *p* < 0.00001 vs. Control). No significant change was observed in the presence of other empty or CE-loaded NP.

### 3.4. Inflammatory Response

**Figure 3:**
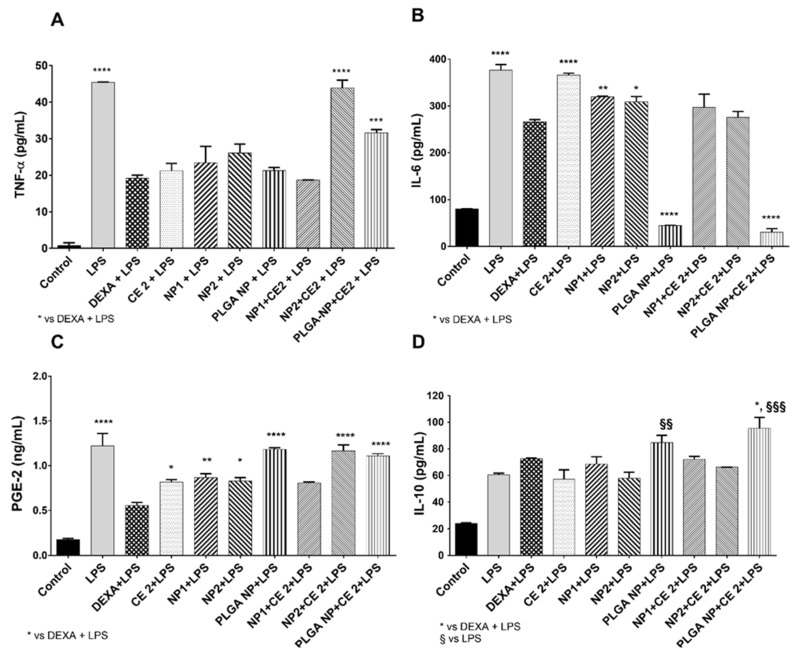
Anti-inflammatory effect of loaded nanoparticles. HUVEC were treated with empty or CE-loaded NPs (NP1:QA-Ch-NP; NP2:QA-Ch-S-pro-NP; and PLGA NP) with 2 µg/mL GAE of CE (CE 2) or DEXA (5 µg/mL) for 2 h and with LPS (10 µg/mL) for 24 h. Evaluation of the inflammatory cytokines secretion: (**A**) TNF-α, (**B**) IL-6, (**C**) PGE2, (**D**) IL-10. Data are expressed as mean ± SD of at least three independent experiments in triplicate. * *p* vs. DEXA + LPS and § *p* vs. LPS. 0.0332(**), 0.0021(***), < 0.0001(****) and 0.0332(§§), 0.0021(§§§).

The literature reports that high concentrations of polyphenols (>10 µg/mL GAE) can reduce the secretion of cytokines in HUVEC [36]. The concentration of 2 µg/mL GAE of total polyphenols was used in the present study on the basis of previous results indicating this concentration as a threshold of CE-loaded NP toxicity on HUVEC [21]. The anti-inflammatory effects of different nanoparticles (NP1:QA-Ch-NP; NP2:QA-Ch-S-pro-NP; and PLGA NPs) loaded with 2 µg/mL GAE of CE were investigated. The secretion of inflammatory cytokines (TNF-α, IL-6, IL-10, PGE2) and nitric oxide production were evaluated in LPS-treated HUVEC by ELISA tests. Figure 3 shows the anti-inflammatory effect of empty or loaded nanoparticles. In particular, CE-loaded NP1 reduced secretion of all pro-inflammatory cytokines (IL-6, TNF-α, and COX-2-dependent prostaglandin E2 (PGE2)), and increased secretion of anti-inflammatory cytokine IL-10 (Figure 3D) at the same level as DEXA, while CE-loaded NP2 and PLGA NPs only reduced IL-6. However, the results observed with PLGA NPs may be due to a more damaging effect on cell viability appearing in Figure 2, where cellular response may reflect the mechanism of a cell attempt to auto-protect from death. Indeed, PLGA NPs induced a significant increase of anti-inflammatory cytokine IL-10 (Figure 3C), correlated with a significant reduction of IL-6 secretion (Figure 3B).

**Figure 4:**
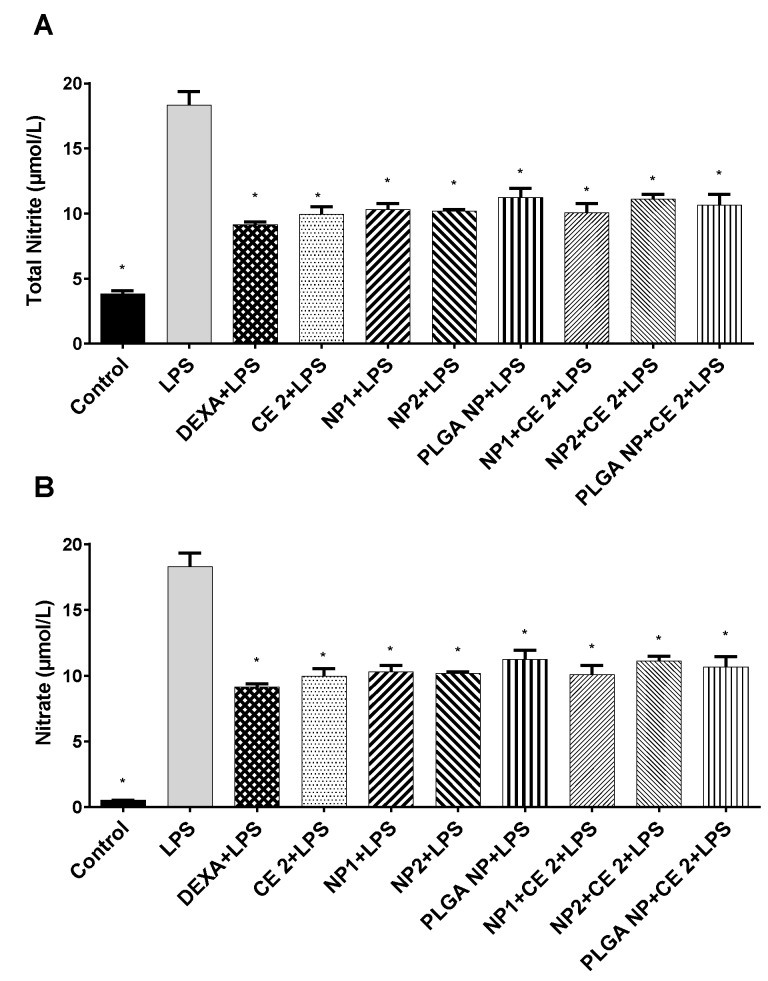
Nitrite (**A**) and nitrate production (**B**). HUVEC were treated with empty or CE-loaded NPs (NP1:QA-Ch-NP; NP2:QA-Ch-S-pro-NP; and PLGA NPs) with 2 µg/mL GAE of CE (CE 2) or DEXA (5 µg/mL) for 2 h and with LPS (10 µg/mL) for 24 h. Data are expressed as mean ± SD of at least three independent experiments in triplicate. * *p* < 0.05 vs. LPS.

Regarding the production of total nitric oxide (NO), the results show that all treatments significantly reduced NO production by HUVEC, when compared with LPS (Figure 4).

### 3.5. NLRP3 Inflammasome Production

**Figure 5:**
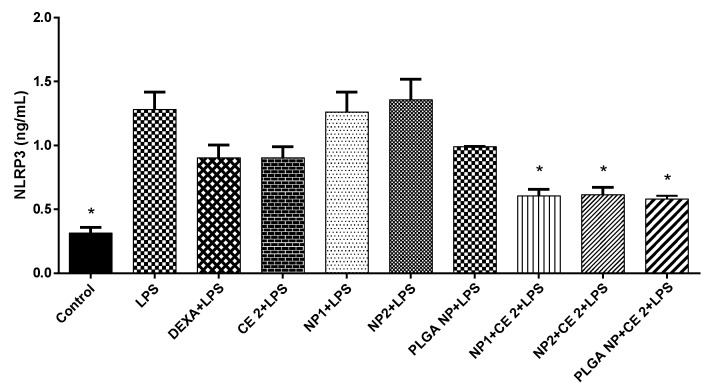
NLRP3 inflammasome activation. HUVEC were treated with empty or CE-loaded NPs (NP1:QA-Ch-NP; NP2:QA-Ch-S-pro-NP; and PLGA NP) with 2 µg/mL GAE of CE (CE 2) or DEXA (5 µg/mL) for 2 h and with LPS (10 µg/mL) for 24 h. Data are expressed as means ± SD of at least three independent experiments in triplicate. * *p* < 0.05 vs. LPS.

To study the potential anti-inflammatory properties of CE-loaded NPs, the secretion of the most known inflammasome NLRP3, a multiprotein scaffolding complex responsible for the activation of inflammatory responses and essential for atherogenesis [37,38,39], was measured. As shown in Figure 5, all types of CE-loaded NPs significantly reduced NLRP3 secretion, thus reducing inflammatory response.

### 3.6. In Vitro Uptake Studies

**Figure 6:**
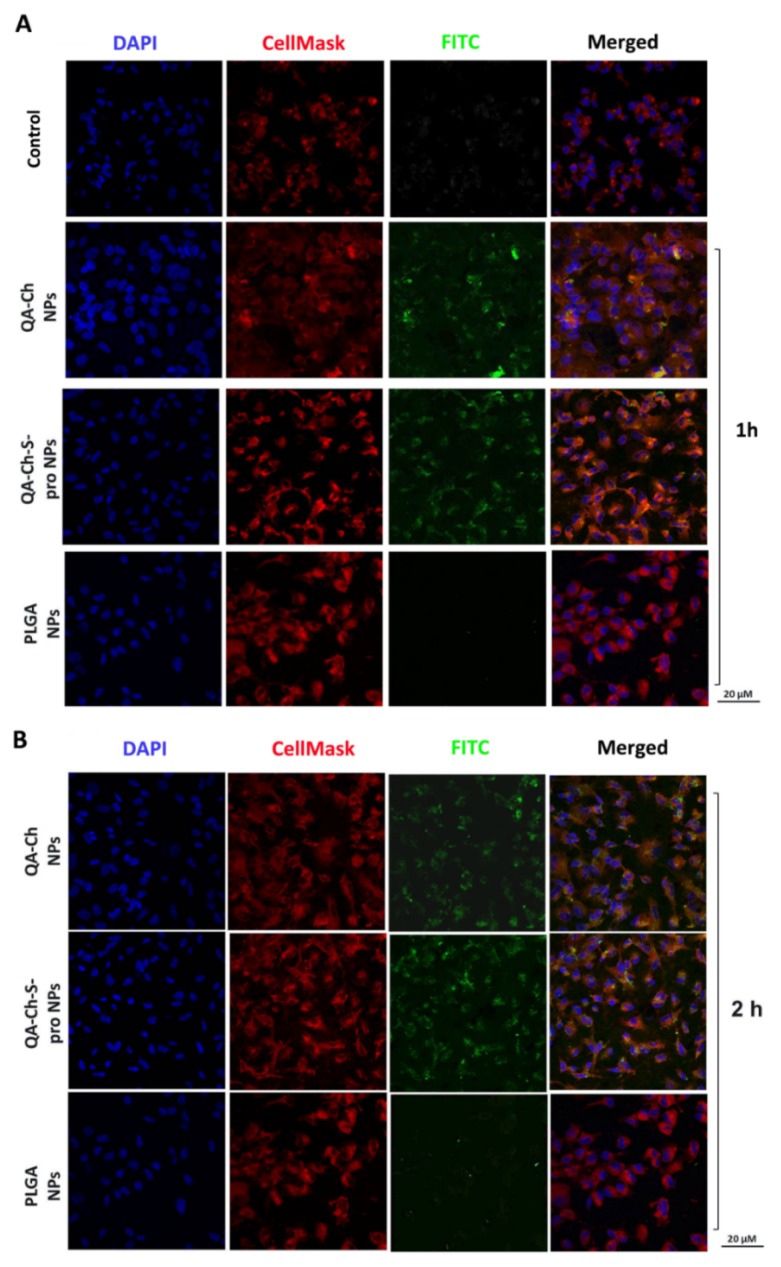
Confocal fluorescence microscopy analysis of HUVEC incubated with FITC-labeled QA-Ch, QA-Ch-S-pro, or PLGA NPs (green color) for 1 h (**A**) or 2 h (**B**). CellMask and 4,6-Diamidino-2-phenylindole (DAPI) for staining the cellular membrane and nucleus are shown in red and blue colors, respectively. Scale bars are 20 µm. Control is represented by untreated cells.

In vitro uptake studies were performed by incubation of HUVEC or C2BBe1 with fluorescent Ch-der or PLGA NPs. CLSM analysis showed that HUVEC were able to internalize the two Ch-der formulations after just 1 h (Figure 6A); however, the accumulation of FITC-labeled NPs in the intracellular membrane was higher after 2 h of treatment (Figure 6B). On the other hand, PLGA NPs analyzed by CLSM were not well visible (Figure 6A,B).

**Figure 7:**
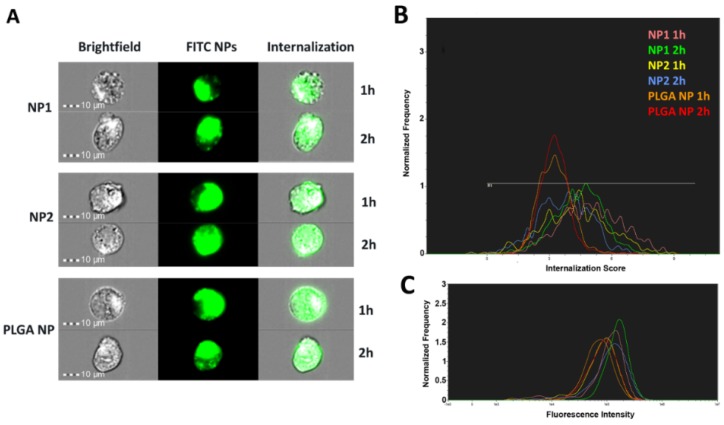
Fluorescence images for FITC-labeled NP internalization by HUVEC (**A**) after 1 h or 2 h treatment, obtained by ImageStream^®^X. (**B**,**C**) The histograms of NP internalization and fluorescence intensity. The respective statistical analysis is reported in Table 2a,b.

To measure fluorescent intensity (FI) of cells, ImageStream^®^X analysis was performed on captured images (Figure 7). As can be seen in Figure 7A, cells show green fluorescence when incubated with either NP1, NP2, or PLGA NPs. In particular, determination of PLGA NP fluorescence was also possible thanks to a higher fluorescence-sensitivity of this laser.

Since the aim of this work was the evaluation of NP anti-inflammatory activity on endothelial cells, we just reported uptake results for HUVEC in the main document. For this reason, all of the uptake results regarding C2BBe1 are presented in Figure S1–S3 and in Table S1a,b. 

### 3.7. NP Quantification and Intracellular Localization

For fluorescence intensity (FI) quantification, the internalization score (IS), which is a measure of the relative amount of signal inside versus outside the cell, was first determined. Positive scores indicate NP internalization by the cells, while negative scores indicate that the NPs are adsorbed on the cell surface. A zero IS value means equal NP adsorption and internalization [32,33]. The IS values are reported in Table 2, where each median value indicates the respective NP amount internalized by cells, regardless of adsorption. An IS value higher than or equal to 3 suggests that HUVEC were able to internalize either Ch-der NPs or PLGA NPs (see Figure 7B), which confirms CLSM results. Data in Table 2 show the highest IS value for NP1. The fluorescence intensity, quantified by the ImageStream^®^X analysis seen in Figure 7C, is reported in Table 3. NP1 shows the highest intensity values after 2 h of treatment (FI: 159675.65 ± 67120.87). PLGA NPs have lower FI values (Figure 7C, Table 3) than Ch-der NPs in HUVEC, as observed by CLSM. Finally, the following rank order of NP interaction with HUVEC was observed: QA-Ch NPs > QA-Ch-S-pro NPs > PLGA NPs.

**Figure 8:**
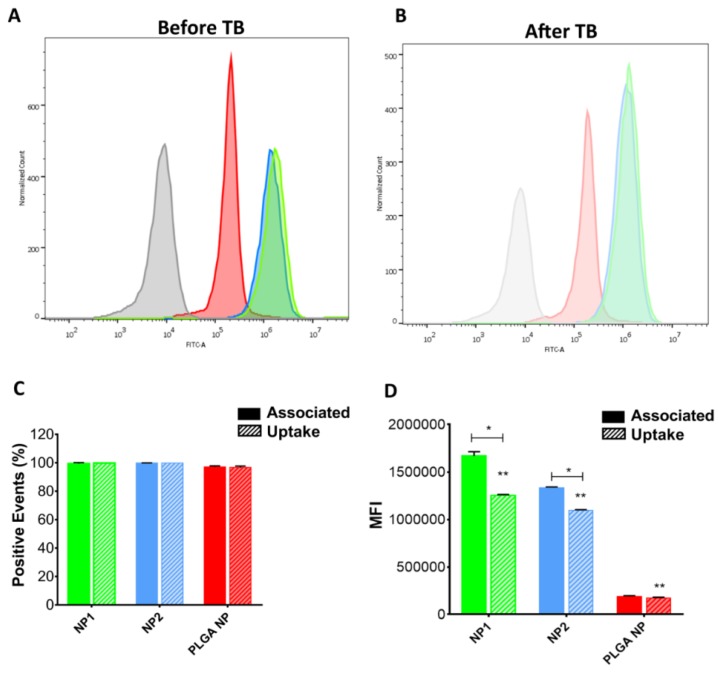
Cytofluorometric analysis. Histograms of association studies between HUVEC and NPs before (**A**) and after (**B**) extracellular fluorescence quenching with trypan blue (TB) by flow cytometry. Control (untreated cells) is represented in grey. (**C**) quantitative determination of cell–NP association and uptake. (**D**) Mean fluorescence intensity (MFI) analysis of extracellular binding and cellular uptake of NPs. Significant differences are indicated with * (*p* < 0.005); ** significant difference between each other.

To study cellular association with NPs and NP uptake, flow cytometry analysis was performed before and after quenching with trypan blue (TB) (Figure 8). The fluorescence intensity of HUVEC incubated for 2 h with NPs shifted considerably to a higher intensity compared to the control, both before and after quenching (Figure 8A,B, respectively). Figure 8C shows that all cells internalized NPs, without differences between the different NP (about 100% of positive events). Nevertheless, the mean fluorescence intensity (MFI) analysis resulted in a reduction after quenching in Ch-der NPs (Figure 8D). In particular, NP1 cellular uptake after 2 h was greater than NP2 or PLGA NP uptake (** *p* < 0.0001), whereas no difference was found between PLGA NPs before and after trypan blue treatment. Moreover, MFI analysis showed a 10-fold (for Ch-der NPs) and a 100-fold (for PLGA NPs) higher value in HUVEC than in C2BBe1 (see Figure S3). 

## 4. Discussion

The aim of this work was to evaluate the anti-inflammatory activity of CE loaded in different types of polymeric NPs on human endothelial cells and the NP ability to increase the bioavailability of polyphenols contained in CE, in order to enhance their beneficial effects.

In a recent study [21], we demonstrated the antioxidant power of Crognola CE and its ability to reduce reactive oxygen species (ROS) production, thanks to its high content in polyphenols. We also showed the ability in vitro of CE-loaded Ch-der NPs and PLGA NPs to enhance the intestinal permeability of polyphenols and maintain their ability to protect HUVEC against oxidative stress [20,21].

In this work, we studied the anti-inflammatory effects of Crognola CE polyphenols, encapsulated in Ch-der NPs (NP1 or NP2) or in PLGA NPs, on HUVEC. In particular, we evaluated the protective effect of CE and NPs against LPS-stressed HUVEC and the production of pro- and anti-inflammatory factors or the inflammasome NLRP3, compared with a strong anti-inflammatory drug, i.e., dexamethasone [40].

First, we assessed NP cytotoxicity and found that cell viability was not affected by any NP type. Then, we studied the CE inflammatory response to LPS treatment by dosing pro- and anti-inflammatory cytokines involved in the inflammatory response [41]. We found that a CE concentration of 2 µg/mL GAE of total polyphenols, when encapsulated in NP1, was effective in inhibiting LPS-induced production of TNF-α and IL-6, and increased the production of IL-10, thus suggesting that CE-loaded NP1 exerts an anti-inflammatory effect, especially on inflammatory mediators and cytokines (see Figure 3). Moreover, we observed that loaded NP2 reduced the IL-6 production induced by LPS, but not TNF-α. Since TNF-α is released earlier than IL-6 [42], this data might be explained by admitting that NP1 are internalized by HUVEC faster than NP2.

Empty or loaded PLGA NPs were unable to protect HUVEC from LPS treatment. This may be due to a PLGA NP detrimental effect on cell viability appearing in Figure 2, where cellular response reflects a protective mechanism from cell death. Indeed, PLGA NPs induced a significant increase of anti-inflammatory cytokine IL-10 correlated with a significant reduction of IL-6 secretion.

We also found that, in all cases, the anti-inflammatory effect of the non-encapsulated CE was either not significantly different or even lower than that of the CE encapsulated in Ch-der NPs (see Figure 3). This indicates that the encapsulated CE maintained its inflammatory activity and probably the free CE and NPs internalization speeds are comparable.

The process of atherosclerosis is associated with a low-grade subclinical inflammation, termed inflammaging, which can accelerate age-related diseases [2]. In Western societies, the age-related inflammatory response can be aggravated by modern lifestyles and excessive calorie consumption [2]. Hence, from here, the importance descends of a fruit-based diet in order to prevent inflammation and related inflammatory factors [3]. It has been reported that cherry consumption can reduce atherosclerosis risk factors [17], and that the NLRP3 inflammasome pathway is centrally involved in the recognition of triggers that appear during physiological aging and metabolic stress [2]. Then it is important to study this protein complex and its interaction with CE. In fact, the silence of NLRP3 causes the stabilization of the atherosclerotic process [39]. In our study we observed a significant NLRP3 inflammasome inhibition by whichever type of CE-loaded NPs, and stressed the importance of cherry in atherosclerosis risk factors prevention. Note that, in the case of NLRP3, the anti-inflammatory effect of loaded NPs was higher than that of DEXA drug (Figure 5).

Moreover, as regards NO and PGE2, which are well-known markers of inflammatory response [43], we demonstrated that CE-loaded NP1 significantly inhibited NO and PGE2 production in LPS-stimulated HUVEC, whereas CE-loaded NP2 did not reduce PGE2 production. This difference could depend on the cells’ ability to internalize the different nanoparticles. For this reason, the interaction of our different NPs with HUVEC, as well as with Caco-2 clone intestinal epithelial cells (C2BBe1) (see Figures S1–S3), was assessed using three different methods, namely, CLSM, ImageStream^®^X, and FACS, in order to better understand their in vitro uptake.

Indeed, the presence of positive charges on the Ch-der NP surface enhance the interaction with the negative charge of membrane and the cytosol phospholipids, which mostly contribute to the negative charge of the cell plasmatic membrane itself [44]. Then, the Ch-der NPs could be more efficiently internalized through an endocytosis clathrin-mediated process rather than a simple endocytosis process, that involves the negatively-charged PLGA NPs [44]. Conversely, any type of NP aggregation during the internalization process could change the size, the shape, or the polydispersity index of the single NP and hence affect, e.g., reduce cellular uptake [44]. This could explain the greater ability of NP1 to be internalized by the HUVEC cells compared to NP2. Indeed, the protected thiol groups on the NP2 surface prevents an early thiol oxidation and allows an exchange reaction with cysteine substructures of the membrane glycoproteins [45], thus forming nanoparticle–glycoprotein aggregates. As Figure 7 shows, NP1 has the highest internalization score and intensity values for HUVEC after 2 h of treatment, pointing to the highest interaction of the positive charges of the QA-Ch polymer with the negative membrane surface. The foregoing arguments could explain the highest anti-inflammatory activity of NP1 on HUVEC.

Particles with a negatively-charged surface, such as PLGA NPs, once internalized by cells, could go through an aggregation/degradation process. This notion, along with the strong dependence of internalization on particle concentration and incubation time, could explain the low NP fluorescence intensity in HUVEC (Figure 6, Figure 7 and Figure 8) [46]. The possibility of PLGA NP adhesion on cell surface could also lead to an alteration of cells metabolic functions and hence explain the cell viability reduction (see Figure 2) [47].

Since both PLGA NPs and Ch-der NPs were internalized in HUVEC after 2 h, the difference in fluorescence intensity could depend on a different interaction with the main proteins that form the membrane of endothelial cells’ tight junctions (TJ) [48]. In fact, previous studies have shown that the mucoadhesivity of nanoparticles, e.g., those based on chitosan derivatives, improves cellular uptake [24,25,49,50]. In fact, Zambito et al. [25] demonstrated that QA-Ch strongly interacted with TJ, and reduced TEER in Caco-2 cell monolayers. This could explain the higher FI value of NP1 than NP2 in C2BBe1 (Figure S2C). However, the higher affinity of all the NP types for HUVEC, demonstrated by the higher internalization score and intensity values (Figure 7 and Figure 8, Figures S2, and S3), can be ascribed to a stronger NP interaction with the TJ of HUVEC, which increases cell permeability. We already demonstrated the ex vivo mucoadhesive properties of Ch-der NPs on rat intestinal mucosa [20]. Therefore, mucoadhesive Ch-der NPs can have a stronger interaction with cells, with respect to non-mucoadhesive PLGA NPs, also thanks to their stronger affinity with TJ, and the presence of a superficial positive charge on the surface which results in a better in vitro uptake and consequently anti-inflammatory activity.

## 5. Conclusions

The results obtained in the present study show that cherry extracts encapsulated in Ch-der and PLGA nanoparticles have good anti-inflammatory activity.

The internalization of CE-loaded NPs in HUVEC has been experimentally demonstrated. The NPs were based on quaternary ammonium chitosan (NP1) or thiolated quaternary ammonium chitosan (NP2) derivatives or poly(lactic-*co*-glycolic acid) NPs. The relevance of internalization to the anti-inflammatory activity of cherry extract loaded in NPs has also been demonstrated. The NP1 uptake in HUVEC after 2 h exposure was greater than that of NP2 or PLGA NPs. This is reflected by the highest anti-inflammatory activity of NP1 on HUVEC and can be ascribed to a higher positive surface charge of NP1.

On the basis of our previous studies, as well as the present one, it was proved that the encapsulation of CE in NPs enhances polyphenols’ anti-inflammatory activity and their intestinal absorption, probably resulting in an improvement in the CE oral bioavailability. In order to obtain a manageable formulation for oral application, these NPs could be lyophilized and introduced into gastro-resistant capsules, which could be regenerated upon contact with the physiologic fluids of the gastrointestinal tract. The study of the lyophilization conditions and the in vivo bioavailability of CE encapsulated in NPs will be the subject of a forthcoming paper.

## Figures and Tables

**Table 1:** Physical characteristics of fluorescein isothiocyanate (FITC)-labeled nanoparticles (NPs).

Nanoparticle Type	Nanoparticle Size, nm	Polydispersity Index	ζ, mV
FITC-QA-Ch NPs (NP1)	191.5 ± 3.7	0.30 ± 0.02	7.0 ± 0.9
FITC-QA-Ch-S-pro NPs (NP2)	235.4 ± 3.2	0.24 ± 0.02	5.5 ± 0.8
FITC-PLGA NPs	201.4 ± 5.7	0.06 ± 0.05	−7.9 ± 0.1

**Table 2:** Fluorescence internalization score (IS) measured by ImageStream^®^X. The IS median values indicate the total amount of NP adsorbed by cells and are correlated to the hystograms reported in Figure 7B. Std. Dev. refers to standard deviation.

Population	Median	Std. Dev.
NP1 1 h	4.961	1.488
NP1 2 h	4.486	1.148
NP2 1 h	4.307	1.545
NP2 2 h	3.754	1.269
PLGA NPs 1 h	3.235	0.8243
PLGA NPs 2 h	3.228	0.7129

**Table 3:** Fluorescent intensity score measured by ImageStream^®^X. The fluorescence median values indicate the amount of NPs really internalized by cells and are correlated to the histograms reported in Figure 7C.

Population	Median	Std. Dev.
NP1 1 h	118,514.27	60,578.46
NP1 2 h	159,675.65	67,120.87
NP2 1 h	86,177.00	51,031.86
NP2 2 h	124,872.98	78,847.77
PLGA NPs 1 h	72,220.92	45,585.81
PLGA NPs 2 h	85,886.73	52,101.06

## Supplementary Materials

The following are available online at https://www.mdpi.com/1999-4923/11/10/500/s1: Figure S1: CLSM analysis of C2BBe1 incubated with FITC-labeled QA-Ch, QA-Ch-S-pro, or PLGA NPs for 1 h (A) or 2 h (B); Figure S2: Fluorescent images of FITC-labeled NP internalization by C2BBe1 after 1 h or 2 h treatment (A), NP internalization score (B), and fluorescence intensity (B) histograms, obtained by ImageStream^®^X imaging flow cytometer; Table S1: Statistical analysis of Figure S1B,C, provided by IDEAS^®^ Software; Figure S3: FACS analysis before (A,C) and after (B,D) fluorescence quenching with trypan blue.
