# Supplementary material for: Anti-Inflammatory Effect of Cherry Extract Loaded in Polymeric Nanoparticles: Relevance of Particle Internalization in Endothelial Cells
Source: Pharmaceutics. 2019 Sep 29;11(10):500. doi: 10.3390/pharmaceutics11100500 (PMC6835553; doi:10.3390/pharmaceutics11100500)
Supplement: Supplementary file 1 [file pharmaceutics-11-00500-s001.pdf]

pharmaceutics

Supplementary Materials: Anti-Inflammatory Effect of Cherry Extract Loaded in Polymeric Nanoparticles: Relevance of Particle Internalization in Endothelial Cells

Denise Beconcini, Francesca Felice, Ylenia Zambito, Angela Fabiano, Anna Maria Piras, Maria Helena Macedo, Bruno Sarmento and Rossella Di Stefano

1. Materials and Methods

1.1. In Vitro Uptake Studies

Caco-2 clone intestinal epithelial cells (C2BBe1) from the American Typical Culture Collection (ATCC, Wesel, Germany) were seeded in 24-well plates (2×10^5 cells/well) on glass coverslips with complete culture medium (Dulbecco's modified Eagle's medium (DMEM) with 4.5 g/L glucose (Lonza) supplemented with 10% FBS (Sigma-Aldrich, St. Louis, MO, USA) penicillin-streptomycin mixture (Gibco, Waltham, MA, USA)) and incubated for 24 h to perform CLSM analysis. The following steps are described in Sections 2.6. and 2.7. of the manuscript.

To perform ImageStream®X and FACS analysis, C2BBe1 were seeded in 6-well plates (3×10^5 cells/well) or 12-well plates (2×10^5 cells per well), respectively, and treated as described in Sections 2.8. and 2.9. of the manuscript.

2. Results and Discussion

2.1. In Vitro Uptake Studies

CLSM analysis (Figure S1) shows that Ch-der NP were able to interact with C2BBe1, at least on the extracellular membrane side, after 1 h treatment (Figure S1A). The NP fluorescence intensity and the quantity of NP that interacted with C2BBe1 are higher after 2 h (Figure S1B), even if lower than those showed by HUVEC (see Figure 6). As in HUVEC, also in C2BBe1 PLGA NP analyzed by CLSM were not well visible, but the fluorescence determination was then possible thanks to a higher fluorescence-sensitivity of ImageStream®X laser. The ImageStream®X analysis reported green fluorescence for all the NP types inside cells (Figure S2A), even if it is less intense respect to the NP fluorescence in HUVEC (Figure 7A).

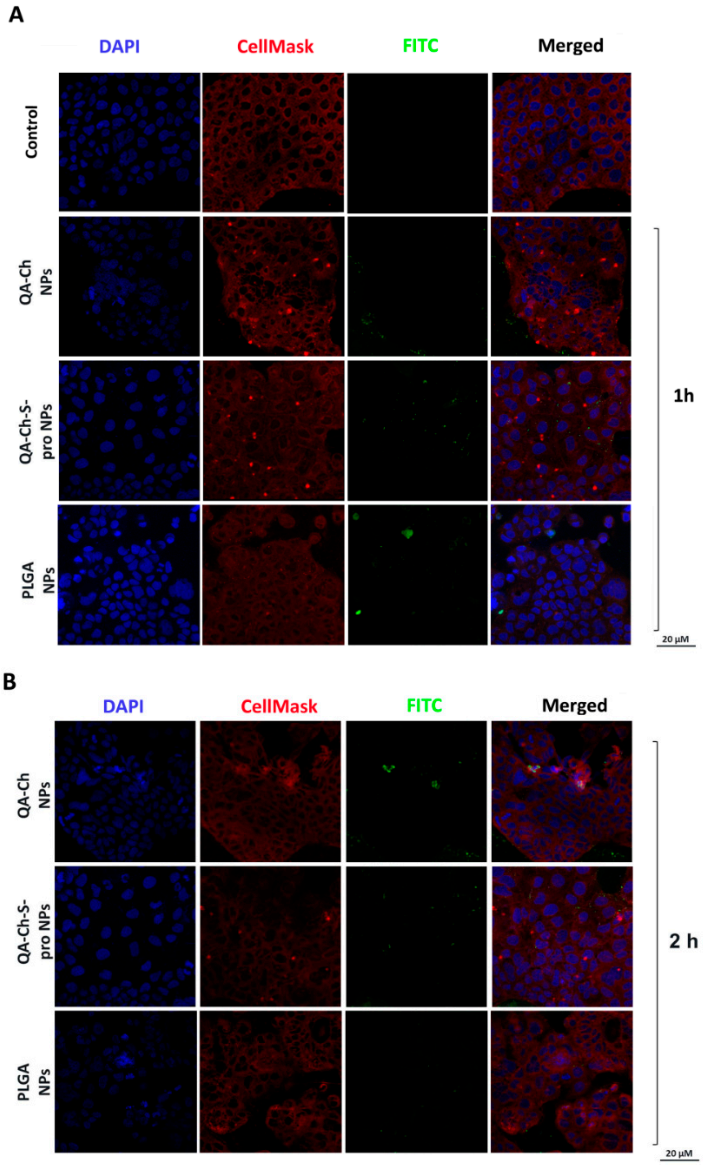

Figure S1. Confocal fluorescence microscopy analysis of C2BBe1 incubated with FITC-labeled QA-Ch, QA-Ch-S-pro or PLGA NP (green color) for 1 h (**A**) or 2 h (**B**). CellMask and DAPI for staining the cellular membrane and nucleus are shown in red and blue colors, respectively. Scale bars are 20 µm. Control is represented by untreated cells.

From the statistical analysis of the histograms showed in Figure S2B, we observe that internalization score (IS) median values (Table S1a) were in all cases higher than 2, meaning that C2BBe1 were able to internalize both Ch-der and PLGA NP. However, IS values are lower than IS values presented by HUVEC (Figure 7B, Table 2a), suggesting that there was probably less interaction of all the NP types with C2BBe1. In particular, this result is confirmed by the fluorescence intensity score of Ch-der NP in C2BBe1 after 2 h (Figure S2C, Table S1b). On the contrary, PLGA NP, which had lower fluorescence intensity values than Ch-der NP in HUVEC, maintain more or less the same values as NP2 in C2BBe1. Finally, NP1 resulted to have the highest IS and intensity score also for C2BBe1 after 2h treatment (Table S1a and S1b).

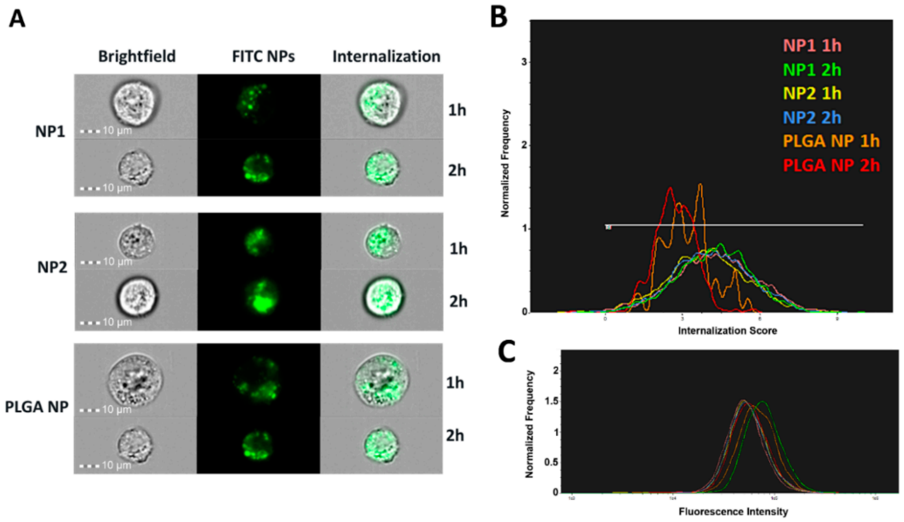

Figure S2. Fluorescence images for FITC-labelled NP internalization by C2BBe1 (**A**) after 1 h or 2 h treatment, obtained by ImageStream®X. Figures **S2B** and **S2C** show the histograms of NP internalization and fluorescence intensity. The respective statistical analysis is reported in **Table S1a** and **S1b**.

Table S1a. Internalization Score.

Population	Median	Std. Dev.
NP1 1 h	4.26	1.608
NP1 2 h	4.28	1.437
NP2 1 h	3.939	1.568
NP2 2 h	4.136	1.491
PLGA NP 1 h	3.167	0.9454
PLGA NP 2 h	2.693	0.82

Table S1b. Fluorescence Intensity.

Population	Median	Std. Dev.
NP1 1 h	50571.67	25584.02
NP1 2 h	76611.38	36767.39
NP2 1 h	53638.81	29585.32
NP2 2 h	53584.42	33999.88
PLGA NP 1 h	66309.91	32415.05
PLGA NP 2 h	56663.53	35704.86

Regarding FACS analysis, we observed that the fluorescence of C2BBe1, incubated for 2 h with the NP, shifted to a higher intensity compared to the control (untreated cells), both before (Figure S3A) and after (Figure S3B) quenching with TB. Figure S3C shows that all cells internalized NP, without differences between the different NP (about 100% of positive events). Nevertheless, the MFI analysis resulted in a reduction after quenching in Ch-der NP (see Figure S3D). In the case of C2BBe1, FACS results report that PLGA NP are uptake as much as NP1 and NP2. However, comparing them with HUVEC (Figure 8D), 10 times (for Ch-der NPs) and 100 times (for PLGA NPs) MFI values lower in C2BBe1 indicate a stronger interaction of all the NP types with HUVEC, as already demonstrated (Section 3.7. of the manuscript).

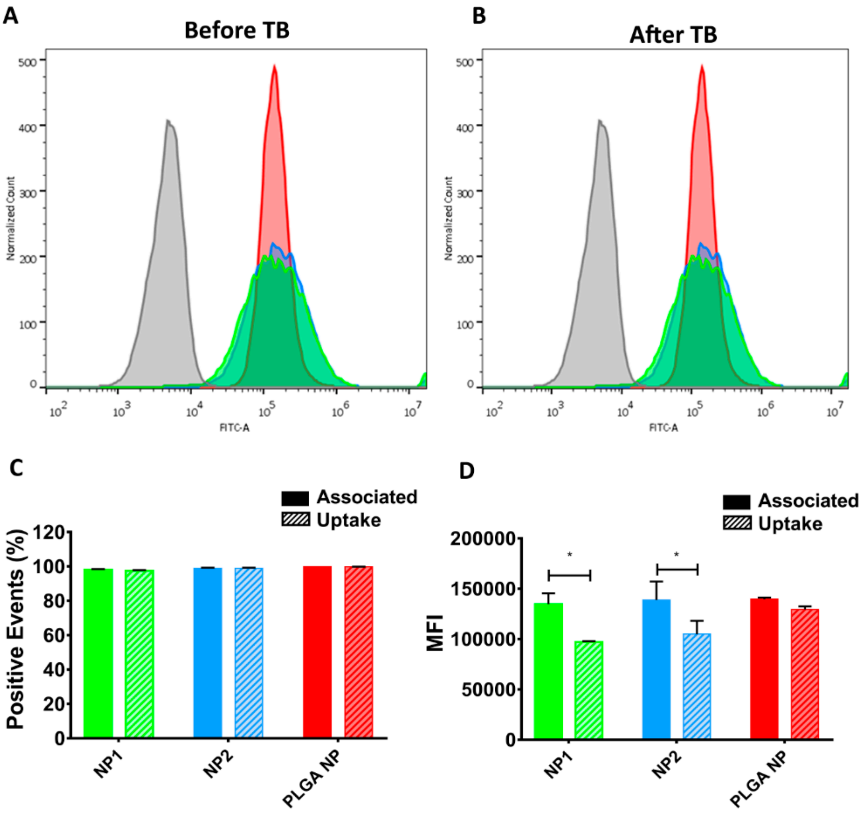

Figure S3. Cytofluorometric analysis. Histograms of association studies between C2BBe1 and NP before (**A**) and after (**B**) extracellular fluorescence quenching with trypan blue (TB) by flow cytometry. Control (untreated cells) is represented in grey. **C**) quantitative determination of cell-NP association and uptake. **D**) Mean fluorescence intensity (MFI) analysis of extracellular binding and cellular uptake of NP. Significant differences are indicated with * ($p < 0.005$).

© 2019 by the authors. Submitted for possible open access publication under the terms and conditions of the Creative Commons Attribution (CC BY) license (http://creativecommons.org/licenses/by/4.0/).
